# Stable Isotope Labeling of Amino Acids in Flies (SILAF) Reveals Differential Phosphorylation of Mitochondrial Proteins Upon Loss of OXPHOS Subunits

**DOI:** 10.1016/j.mcpro.2021.100065

**Published:** 2021-02-25

**Authors:** Florian A. Schober, Ilian Atanassov, David Moore, Javier Calvo-Garrido, Marco F. Moedas, Anna Wedell, Christoph Freyer, Anna Wredenberg

**Affiliations:** 1 Department of Molecular Medicine and Surgery, Karolinska Institutet, Stockholm, Sweden; 2 Max Planck Institute Biology of Ageing - Karolinska Institutet Laboratory, Division of Metabolic Diseases, Department of Laboratory Medicine, Karolinska Institutet, Stockholm, Sweden; 3 Proteomics Core Facility, Max Planck Institute for Biology of Ageing, Cologne, Germany; 4 Department of Medical Biochemistry and Biophysics, Karolinska Institutet, Stockholm, Sweden; 5 Centre for Inherited Metabolic Diseases, Karolinska University Hospital, Stockholm, Sweden

**Keywords:** Drosophila melanogaster, SILAC, post-translational modification, phosphorylation, metabolism, mitochondria, LRPPRC, NDUFA4, NDUFB10, ACN, acetonitrile, adj, adjusted, bsf, bicoid stability factor, cryo-EM, cryogenic electron microscopy, dae, days after egglay, *Dm*, *Drosophila melanogaster*, FC, fold change, FDR, false discovery rate, GSEA, gene set enrichment analysis, GuCl, guanidinium chloride, *Hs*, *Homo sapiens*, KD, knockdown, KEGG, Kyoto Encyclopedia of Genes and Genomes, LFQ, label-free quantification, LRPPRC, leucine-rich pentatricopeptide repeat motif-containing protein, Lys-6, L-lysine-[^13^C_6_], ORA, overrepresentation analysis, OXPHOS, oxidative phosphorylation, PEPCK, phosphoenolpyruvate carboxy kinase, PKA, protein kinase A, PTM, posttranslational modification, SILAC, stable-isotope labeling of amino acids in cell culture, SILAF, stable-isotope labeling of amino acids in flies, TCA, tricarboxylic acid, TiO_2_, titanium dioxide, *wDah*, white Dahomey

## Abstract

*Drosophila melanogaster* has been a workhorse of genetics and cell biology for more than a century. However, proteomic-based methods have been limited due to the complexity and dynamic range of the fly proteome and the lack of efficient labeling methods. Here, we advanced a chemically defined food source into direct stable-isotope labeling of amino acids in flies (SILAF). It allows for the rapid and cost-efficient generation of a large number of larvae or flies, with full incorporation of lysine-[^13^C_6_] after six labeling days. SILAF followed by fractionation and enrichment gave proteomic insights at a depth of 7196 proteins and 8451 phosphorylation sites, which substantiated metabolic regulation on enzymatic level. We applied SILAF to quantify the mitochondrial phosphoproteome of an early-stage leucine-rich PPR motif-containing protein (LRPPRC)-knockdown fly model of mitochondrial disease that almost exclusively affects protein levels of the oxidative phosphorylation (OXPHOS) system. While the mitochondrial compartment was hypo-phosphorylated, two conserved phosphosites on OXPHOS subunits NDUFB10 and NDUFA4 were significantly upregulated upon impaired OXPHOS function. The ease and versatility of the method actuate the fruit fly as an appealing model in proteomic and posttranslational modification studies, and it enlarges potential metabolic applications based on heavy amino acid diets.

## Highlights

• Adaptation of a fully defined, holidic food source for fruit fly SILAC (SILAF)
• More than 99% labeling efficiency with lysine-6 within one larval generation
• One milligram of fully lysine-6 labeled peptides for less than $15
• 14,000 *Drosophila* phosphorylated sites with 1500 occupancy values
• Mitochondrial DmLRPPRC1 loss increases phosphorylation of NDUFB10 and NDUFA4

## Graphical abstract

**Figure:**
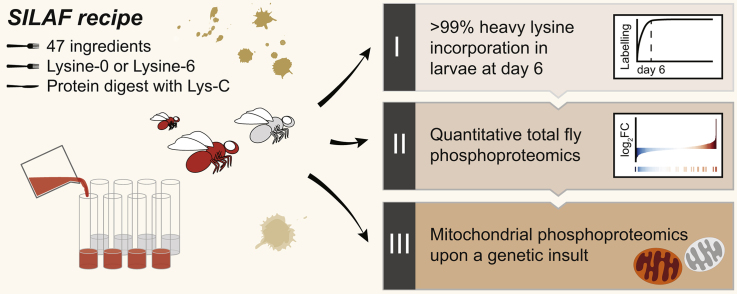

## In Brief

We advanced a fully composed food source for *Drosophila melanogaster* into a highly efficient, versatile, and cheap labeling method, termed SILAF. The larval proteome incorporates more than 99% heavy lysine-6 label within 6 days, while the adult fly metabolism allows protein turnover studies. We obtained a phosphoproteome with site-specific occupancy in a fly model of mitochondrial metabolic disease, highlighting the regulation of two novel conserved phosphosites on subunits of the electron transport chain.

Stable-isotope labeling of amino acids in cell culture, known as SILAC, uses nonradioactive isotopic labeling to detect differences in relative abundance between at least two peptide populations simultaneously (1). This allows accurate peptide and protein quantification even upon enrichment or fractionation of peptide mixtures and thus greatly enhances the depth of large-scale screening for proteins, posttranslational modifications (2) or protein–protein interactions (3). The simplicity and robustness of SILAC have led to its broad adoption in a variety of biological specialties and have been applied to a number of animal models (4).

The fruit fly *Drosophila melanogaster* (*Dm*) is most commonly raised on food sources composed of metabolically undefined mixtures such as yeast and cornmeal. The first SILAC method in the fly was based on lysine auxotrophic yeast cultured in medium with a stable lysine isotope (5). Although technically feasible, high prospective costs for stable isotopes (6), a general inflexibility concerning further heavy amino acids due to yeast metabolism, and the labeling efficiency of at most 96.7% just above the threshold for SILAC usability have prevented a wide adoption of the method. Systematic evaluation indicated undesirable conversion of heavy amino acids, suggesting an underestimation of the heavy to light ratio on protein level (6). Although normalization on protein level can account for this, these limitations rendered the SILAC fly inaccessible for *Drosophila* laboratories not specialized on proteomics data analysis. Thus, signal-critical applications such as quantification of posttranslational modifications (PTM) have not been widely performed with SILAC in fly models, leaving the vast number of genetic *Drosophila* tools and fly stock libraries mostly unused in the proteomics era.

PTMs of the proteome are vital switches to establish and maintain the adaptive capacity of cells including reactions to metabolic insults (7). Mitochondria form a central hub in cellular metabolism and must rapidly respond to changing physiological demands. It is therefore feasible to suggest that PTMs play an important role in this regulation. Approximately 1200 proteins reside in mitochondria (8); 13 are encoded on mitochondrial DNA with translation occurring inside mitochondria, while the vast majority are synthesized on cytosolic ribosomes and imported *via* designated protein import and sorting pathways. A key function of mitochondria is the production of ATP *via* the oxidative phosphorylation system (OXPHOS). Although OXPHOS structure has been described, assembly and regulation are incompletely understood. A number of kinases and phosphatases are localized to the mitochondrial matrix (reviewed in ref. (9)), and both metabolic regulators and protein components of OXPHOS are differentially phosphorylated in obese mice (7, 10). However, a direct dependency of mitochondrial protein phosphorylation status on OXPHOS integrity has not been assessed yet. Phosphorylation sites were found underrepresented on mitochondrial proteins in a human cell line (11) and mice (10) compared with other cellular compartments. Thus, precise and deep quantitative phospho-proteomics are imperative to understand the functional and structural role of mitochondrial PTMs in response to changing cellular demands and disease.

Here we advanced a chemically defined food source, allowing direct stable-isotope labeling of amino acids in flies (SILAF) and enabling proteomic analysis at subcellular resolution. We applied SILAF to quantify the mitochondrial phosphoproteome of an early-stage leucine-rich PPR motif-containing protein (LRPPRC)-knockdown model of mitochondrial disease that exclusively affects the intramitochondrial synthesis of proteins involved in oxidative phosphorylation (OXPHOS). Among a total library of more than 14,008 fly phosphorylation sites, we identify 33 quantifiable sites on subunits of the mitochondrial respiratory chain with two of these sites, on NDUFB10 and NDUFA4, showing regulatory properties upon impaired OXPHOS function.

## Experimental Procedures

### Experimental Design and Statistical Rationale

The overall experimental design consist of: (1) validating incorporation rates of stable-isotope labeled amino acids in larvae and flies; (2) correlating the proposed direct SILAF method with an established indirect SILAC technique in *Dm*, further testing its feasibility for small-scale peptide fractionation; (3) assessing SILAF performance in combination with large-scale peptide fractionation with or without phosphopeptide enrichment; (4) applying the technique to a biological question of significance by quantifying phosphosite occupancies on mitochondrial proteins upon loss of core OXPHOS subunits. All experiments are biologically replicated, and the number of replicates for each experiment is stated in the corresponding figure legend, including statistical tests used. An equal number of controls was used in each experiment, the type of control is described in each results section. In addition to a biological replicate, phospho-enriched samples were analyzed using two technical replicates.

### Fly Stocks and Husbandry

Experiments were performed on a white Dahomey (*wDah*) fly line (a gift from Professor Linda Partridge at the Max-Planck Institute for Biology of Ageing, Cologne, Germany). Ubiquitous knockdown of *bsf/DmLRPPRC1* was achieved by crossing the previously published fly line *w;;UAS-bsfRNAi#1* (12) to a daughterless-GAL4 driver line. All stocks were backcrossed into the *wDah* genetic background for at least six generations. Parental flies were put to lay for 8 h at an approximate density of 50 males to 50 females on either yeast or holidic medium. Experiments were performed on a mix of male/female stage three larvae if not indicated differently, or on 1-day-old male flies to eliminate the bias of egg-carrying female flies. Flies were maintained at 25 °C and 60% humidity on a 12 h:12 h light:dark cycle.

### Holidic and Yeast Medium

Yeast medium was prepared with a standard yeast–sugar–agar composition (supplemental Table S1). Holidic medium was prepared as described by Piper *et al.* (13, 14). Working reagents are summarized in supplemental Table S1. The FLYAA composition was followed for amino acid ratios. For SILAF labeling, L-lysine was substituted as appropriate with either light L-lysine-[^12^C_6_] HCl (Sigma-Aldrich, L8662) or heavy L-lysine-[^13^C_6_, ^15^N_0_] HCl (Sigma-Aldrich, 643459) and L-Arginine was substituted as appropriate with either light L-arginine-[^12^C_6_,^14^N_4_] HCl (Sigma-Aldrich, A5131) or heavy L-arginine-[^13^C_6_,^15^N_4_] HCl (Sigma-Aldrich, 608033). Medium was stored for up to 10 days at 4 °C. All procedures were performed using semisterile techniques.

### Mitochondrial Enrichments from Drosophila Larvae

Fly larval mitochondria were prepared by disrupting larvae with a 15 ml Teflon-coated dounce homogenizer at 14 strokes and 700 rpm in STE buffer (250 mM sucrose, 5 mM Tris-HCl, 2 mM EGTA, pH 7.4) with 5% BSA lyophilized powder. Mitochondria were enriched by differential centrifugation at 4 °C first at 1000*g* for 5 min and 3000*g* for 10 min. The pellet was washed twice with STE buffer without BSA and mitochondria were further purified at 3000*g* for 10 min and 7000*g* for 10 min. Protein content was determined using Qubit protein assay reagents (Thermo Fisher Scientific). The purity of mitochondria that this method yields was reported by Pajak *et al.* (15).

### Cell Culture and Mitochondrial Enrichment

HEK 293T cells were grown at 37 °C, 60% humidity, 5% CO_2_ in Dulbecco’s modified Eagle’s medium high glucose, GlutaMAX (Thermo Fisher Scientific) supplemented with 10% fetal bovine serum (Thermo Fisher Scientific), 1% penicillin/streptomycin (Thermo Fisher Scientific). HsNDUFA4-FLAG and HsNDUFA4-S66A-FLAG cDNA sequences (GenScript, custom order) were cloned into the retroviral pBABE-Puromycin vector by Gateway cloning (Thermo Fisher Scientific). Phoenix amphotropic cells were transfected with the pBABE constructs using Lipofectamine 3000 (Thermo Fisher Scientific) following manufacturer’s instructions. Retrovirus-containing medium was filtered (0.45 μm, Sarstedt) and added to HEK293T cells together with 4 μg/ml polybrene. Stably transduced cells were cultured as described above while maintaining under puromycin selection (1.5 μg/ml). Cells were immortalized by transient expression of SV40 large T antigen (addgene plasmid #21826). For experiments, cells were grown to about 80% confluency, medium was removed, and cells were washed once in PBS. Mitochondria were prepared as described in ref. (16), following the procedure for mouse embryonic fibroblasts. Mitochondria were aliquoted and frozen in liquid nitrogen, before being stored at −80 °C.

### Blue Native PAGE

Fifty micrograms of mitochondria was resuspended in lysis buffer composed of 1% digitonin, 0.1 mM EDTA, 50 mM NaCl, 10% glycerol, 1 mM PMSF, 2 mM Tris-HCl, pH 7.4, and incubated on ice for 10 min. Detritus was pelleted for 10 min at 12,000*g* and the supernatant was mixed with 10x sample buffer (5% brilliant blue G, 10 mM Bis-Tris, pH 7.0, 500 mM aminocaproic acid). A total volume of 20 μl was loaded on a 3% to 12% NativePAGE gel (Thermo Fisher Scientific), using NativePAGE buffer (Thermo Fisher Scientific). Complexes were first separated at 60 V for 2 h, then the cathode buffer was changed from dark to light blue, followed by 150 V for 1 h. Gels were then incubated in transfer buffer (20% methanol, 0.1% sodium dodecyl sulfate, 190 mM Glycine, 25 mM Tris-HCl, pH 8.3) for 2 h. Gels were transferred to PVDF membranes over night at 25 V. Membranes were then rinsed in pure methanol for 30 s to remove Coomassie dye, blocked for 1 h in TBS-T (140 mM NaCl, 25 mM Tris-HCl, pH 7.4, 0.1% Tween-20) with 5% milk powder. Primary antibodies were anti-DDDK (abcam, ab1257) and total OXPHOS human WB antibody cocktail (ab110411) from Abcam. Secondary antibodies were coupled to HRP, and Clarity Western ECL (Biorad) was used for developing.

### Peptide Preparation

Larvae were collected by either picking them with a sterile inoculation needle or by rinsing the food with distilled water. Larvae were cleaned in distilled water, soaked dry, collected in 1.5 ml low-binding tubes (Eppendorf, 0030108116), and snap-frozen in liquid nitrogen. Flies were collected by a short carbon dioxide anaesthesia, collected in low-binding tubes, and snap-frozen in liquid nitrogen. For all experiments with the exception of the phosphoproteomes, ten insects were used as either a random mix of female/male larvae or as male flies only if not stated differently. SILAF samples were thus a mix of five light and five heavy insects. All samples were stored at −80 °C before processing.

For protein extraction, samples were covered with 200 μl 6 M guanidine hydrochloride (GuCl, Sigma-Aldrich G3272) in 20 mM Tris-HCl, pH 8.0. All samples were homogenized with a Teflon pestle for about 10 s. Samples were incubated at room temperature for 10 min, then sonicated at a 10 s on/off cycle in ice-cold water four times, followed by a further incubation of 10 min at room temperature. A centrifugation step for 5 min at maximum speed pelleted most of the insoluble chitin, although a floating layer can occur. In total, 180 μl of supernatant without any visible fly chitin was transferred to a new 1.5 ml low-binding tube. An aliquoted 1:10 dilution in water was quantified with Pierce BCA protein assay (Thermo Fisher Scientific Cat. No. 23225). While the protein assay was incubating at 37 °C in the dark, proteins were first reduced for 30 min at 55 °C with 5 mM dithiothreitol, cooled down briefly on ice, and then alkylated for 15 min at room temperature in the dark with 15 mM 2-chloroacetamide (17). In total, 50 μg of each light and heavy protein sample was then mixed, transferred to a new 2 ml low-binding tube, and brought to 1.8 ml with 110 mM Tris-HCl pH 8.5. Label-free samples were used at 100 μg. Either Pierce lysyl endopeptidase LysC, MS grade (Thermo Fisher Scientific Cat. No. 90307) or Pierce trypsin, MS grade (Thermo Fisher Scientific Cat. No. 90057) was added at 1:50, equivalent to 2 μg of protease and incubated horizontally over night for a maximum of 18 h at 37 °C and mild shaking.

Samples were acidified with 1.2% formic acid, spun at 3000*g* and room temperature for 10 min, and desalted on 3 ml EmporeTM SPE cartridges (Sigma-Aldrich 66872-U), following the manufacturer’s recommendations. In brief, the column was conditioned with 200 μl methanol and equilibrated with 500 μl water. The sample was loaded followed by 1 ml acidified sample buffer and two washes of 500 μl each, the first one with water, the second one with 0.5% formic acid. The peptides were eluted off the column with 300 μl of 40% acetonitrile two times and lyophilized in a SpeedVac (Savant SC110A vacuum concentrator with refrigerated vacuum trap). Peptides were resolubilized in 20 μl of 0.5% formic acid and quantified on a NanoDrop 1000 spectrophotometer at 280 nm. Following this protocol, anticipated amounts are between 20 and 35 μg of peptides upon 100 μg protein input. Peptides were dried again, stored at −80 °C, and sent for mass spectrometry analysis on dry ice.

For the adult phosphoproteome, 250 male flies were collected for each labeling state. 860 mg GuCl was added and brought to 1.5 ml with 20 mM Tris-HCl pH 8.0 to obtain a final concentration of 6 M GuCl. Homogenization was performed as described above and 1.3 ml was transferred to a 15 ml tube. Reduction and alkylation were performed as described above and samples were diluted 1:10, which obtained about 1 mg/ml. Mitochondria were prepared from three vials of developmentally matched larvae per genotype and replicate as described above, except for addition of one tablet PhosSTOP (Roche) per 10 ml of buffer. Mitochondria were snap-frozen as pellets in liquid nitrogen and stored at −80 °C. The mitochondrial pellets were then resuspended in 500 μl 6 M GuCl and reduced and alkylated as described above. 10 milligrams of adult protein extract or 3 mg of mitochondrial protein from each sample was combined and subjected to digestion by 40 μg or 10 μg endoproteinase LysC at 37 °C for 12 h. Next, 10 μg or 2 μg of LysC was added and the digestion was carried out for subsequent 4 h. Peptide samples were acidified using formic acid to a final concentration of 1% and centrifuged at 2400*g* for 10 min. The supernatant, containing peptides, was desalted by solid-phase extraction using C18 Sep-Pak cartridge, 200 mg (Waters, WAT054945). The cartridge was conditioned with 1 ml methanol, 1 ml 60% acetonitrile, 1% formic acid, and twice with 1 ml 1% formic acid. Next sample was applied and washed twice with 2 ml 1% formic acid. Finally, the peptides were eluted using 600 μl 60% acetonitrile, 1% formic acid dried in a vacuum centrifuge. For large pH reversed-phase separation, the peptide pellet was dissolved in 50% ACN, 0.01 M using 10 min sonication in a water bath. The solution was diluted to 5% ACN, 0.01 M ammonium bicarbonate, centrifuged for 10 min at 20,000*g* on a tabletop centrifuge, and 95% of the solution was separated by large-scale high pH reversed-phase separation.

### Small-scale High pH Reversed-phase Separation

Twenty micrograms of peptides derived from the SILAF comparison of male and female fly were separated on a 150 mm, 300 μm, 2 μm C18, Acclaim PepMap (Product No. 164537 Thermo Fisher Scientific) column using a Ultimate 3000 (Thermo Fisher Scientific). The column was maintained at 30 °C. Buffer A was 5% acetonitrile 0.01 M ammonium bicarbonate, buffer B was 80% acetonitrile 0.01 M ammonium bicarbonate. Separation was performed using a segmented gradient from 1% to 50% buffer B, for 85 min and 50% to 95% for 20 min with a flow of 4 μl. Fractions were collected every 150 s and combined into nine fractions by pooling every ninth fraction. Pooled fractions were dried in Concentrator plus (Eppendorf), resuspended in 5 μl 0.1% formic acid from which 2 μl was analyzed by LC-MS/MS.

### Large-scale High pH Reversed-phase Separation and Phosphopeptide Enrichment

Peptides derived from each biological replicate were separated on a 4.6 x 250 mm ZORBAX 300 Extend-C18, 5 μm, column (Agilent Technologies) at a 1 ml flow rate using a NGC Quest 10 chromatography system (Bio-Rad). Buffer A was 5% acetonitrile 0.01 M ammonium bicarbonate, buffer B was 80% acetonitrile 0.01 M ammonium bicarbonate. Buffers were prepared with LC-MS grade water. Peptide separation was performed using a segmented gradient from 5% to 27% buffer B for 65 min and from 27% to 45% buffer B for 30 min. Eluting peptides were collected for 72 min using a BioFrac fraction collector (Bio-Rad). Fraction collection pattern was set to row and fraction collection size was set to 0.75 ml. In total, 96 fractions were collected in a 1.2 ml V-shaped 96-well plate (Biotix). Upon sample collection, plates were dried overnight in Concentrator plus (Eppendorf) and peptides were resuspended in 20 μl 80% acetonitrile, 2% trifluoroacetic acid. All 96 fractions were concatenated into 12 fractions for adult total fly peptides or eight fractions for mitochondrial larval peptides by combining every 12th or eighth fraction. One-tenth of the volume was dried, resuspended in 5 μl 0.1% formic acid from which 2 μl was analyzed by LC-MS/MS. The remaining solution was diluted with lactic acid (GL Sciences) and phosphopeptides were enriched with 3 mg TiO_2_ tips (GL Sciences) using the manufacturer’s instructions. Enriched phosphopeptides samples were dried and resuspended in 5 μl 0.1% formic acid from which 2 μl was used for analysis by LC-MS/MS.

### LC-MS/MS Analysis

Peptides were separated on a 25 cm, 75 μm internal diameter PicoFrit analytical column (New Objective) packed with 1.9 μm ReproSil-Pur media (Dr Maisch) using an EASY-nLC 1200 (Thermo Fisher Scientific). The column was maintained at 50 °C. Buffers A and B were 0.1% formic acid in water and 80% acetonitrile, 0.1% formic acid, respectively. Peptides were separated on a segmented gradient from 6% to 31% buffer B for 82 min and from 31% to 44% buffer B for 5 min at 200 nl/min. Eluting peptides were analyzed on a QExactive HF mass spectrometer (Thermo Fisher Scientific). Peptide precursor mass-to-charge ratio (m/z) measurements (MS1) were carried out at 120,000 resolution in the 350 to 1500 m/z range. The top seven most intense precursors, with charge state from 2 to 6 only, were selected for HCD fragmentation using 27% normalized collision energy. The m/z of the peptide fragments (MS2) was measured at 30,000 resolution, in profile mode, using an AGC target of 2e5 and 80 ms maximum injection time. Upon fragmentation precursors were put on an exclusion list for 45 s.

Phosphopeptide-enriched samples were analyzed using two technical replicates. For the first replicate, peptides were separated with a gradient from 6% to 31% buffer B for 70 min and from 31% to 44% buffer B for 17 min, using MS parameters as described above. For the second replicate, peptides were separated with a gradient from 6% to 31% buffer B for 53 min and from 31% to 44% buffer B for 4 min. The MS parameters were as described, except that the MS2 of the top five most intense precursors was measured at 60,000 resolution with 110 ms injection time. The mitochondrial larval peptides and phosphopeptides were separated on a 75 cm, 75 μm internal diameter C18 Acclaim PepMap column (Thermo Fisher Scientific); the phosphopeptides were analyzed using two technical replicates. For the second technical replicate, the exclusion time was set to 5 s.

Peptide fractions from the small-scale high pH reversed-phase separation were analyzed on an Orbitrap Tribrid Fusion (Thermo Fisher Scientific) with the same MS parameters as described above, except for centroid mode of acquisition in MS2.

### Quantification and Statistical Analyses

Raw data were mapped in batches for either female/male comparison, the holidic phosphoproteome and proteome, quality control and proline-6 conversion or *DmLRPPRC1* KD with MaxQuant version 1.6.1.0 (18) against the canonical and isoform sequences of the fruit fly proteome (UP000000803_7227 from UniProt, downloaded in September 2018 with 21,939 entries). The reference database was complemented by MaxQuant’s own default contaminant library. Methionine oxidation and N-terminal acetylation were set as variable modifications, cysteine carbamidomethylation as a fixed modification. The maximum number of modifications per peptide was set to five. *In silico* digestion was performed with LysC/P as a protease with maximum two missed cleavages. A false discovery rate of 0.01 was chosen for protein identification and for peptide spectra matches. Database searching was done using a tolerance of 20 ppm for the first search, and 4.5 ppm as main search tolerance. Label-free samples were quantified with default LFQ settings.

Quality control of the mapped proteinGroup.txt file was performed additionally for each study in Perseus version 1.5.0.0 (18). Normalized H/L ratios or label-free values were imported for each sample and proteins marked as “reverse,” “only identified by site,” and “potential contaminant” were excluded. Incorporation rates were calculated from not-normalized intensities by: Intensity Heavy/(Intensity Heavy + Intensity Light). Fold changes between two fractions were derived from normalized SILAC Heavy/Light ratios. For phosphoscreens, phosphorylation on STY was additionally set as a variable modification. Phosphorylation sites were filtered for localization probability of >0.75; occupancies per replicate were sites per replicate calculated as occupancy Heavy/occupancy Light and are presented as log_2_-transformed values. LFQ values were imported and mean values were calculated on not-transformed data. For better visualization, LFQ intensities were log_2_ and *p* values log_10_ transformed.

The proteinGroup.txt file and the Perseus output were imported into R version 3.5.2 running in an RStudio environment, version 1.1.383. The R code is publicly available *via* GitHub: https://github.com/Zinksulfid/SILAF_public.git. Figures were plotted with ggplot2 version 2.2.1.9000 and extension packages, see the original script. Adjusted *p* values for plotting were calculated as FDR with limma moderated *t* test (19), which is part of the limma package version 3.38.3. The *Dm*-specific MitoXplorer (20) library was used to annotate mitochondrial proteins and the functional processes they are related to.

Gene set enrichment analyses (GSEA) and overrepresentation analyses (ORA) were performed with the WebGestalt 2019 online application (21) by uploading a ranked file containing Entrez Gene identifiers and mean log_2_-fold changes. Uniprot IDs were converted to Entrez Gene IDs using the package UniProt.ws version 2.16.0 or all other identifiers using org.Dm.eg.db version 3.8.2 in R. Mouse orthologs of fly OXPHOS subunits were identified with DIOPT-DRSC Integrative Ortholog Prediction Tool (22). Multiple sequence alignment was done with the web interface of Clustal Omega (23). Previously identified human phosphorylation sites were retrieved from PhosphoSitePlus (24).

A description of statistical parameters used in each experiment is provided in the figure legends.

### Structural Modeling

Structures were visualized in PyMOL 2.3.0, for which *Homo sapiens* cryo-EM-derived structures of complex I (EMD-6772 and 5XTC) (25) and complex IV (5Z62) (26) were used. The cryo-EM density mesh is represented at a contour level of 10.0 and carve of 2. Phosphorylated residues were modeled with the PyTMs plugin (27) with optimization level set to 1, local radius 10 Å, interval 30, states 0, and removal radius 5.0.

## Results

### SILAF Enables Full Amino Acid Labeling

To overcome the difficulties associated with traditional yeast-based food in SILAC, we used a holidic food source in this study containing 47 ingredients (13, 14), including defined amounts of eight essential amino acids. In its more popular application in cell culture, SILAC relies on labeling with both “heavy” lysine and arginine followed by digestion with trypsin, which cuts C-terminally of both amino acids. However, previous results in both cells and the fly suggested that arginine can be metabolized into other amino acids (6, 28). In agreement with this, growing larvae on holidic food containing heavy ^13^C_6_-lysine and ^13^C_6_^15^N_4_-arginine, followed by tryptic peptide preparation and liquid chromatography tandem mass spectrometry (LC-MS/MS), identified a number of heavy [^13^C_5_^15^N_1_] proline-containing peptides (supplemental Fig. S1, *A* and *B*). In addition, we observed a decrease in the average intensity of the heavy fraction in an equal mass peptide mixture from heavy and light holidic food (supplemental Fig. S1*C*). We therefore switched to a holidic medium containing only heavy lysine and performed protein digestion with the endoproteinase Lys-C. This equalized heavy and light intensities (supplemental Fig. S1*C*), supporting the absence of metabolic turnover and incorporation into other amino acids. Reassuringly, the usage of Lys-C as opposed to trypsin did not compromise summed protein intensities (supplemental Fig. S1*D*).

**Fig. 1:**
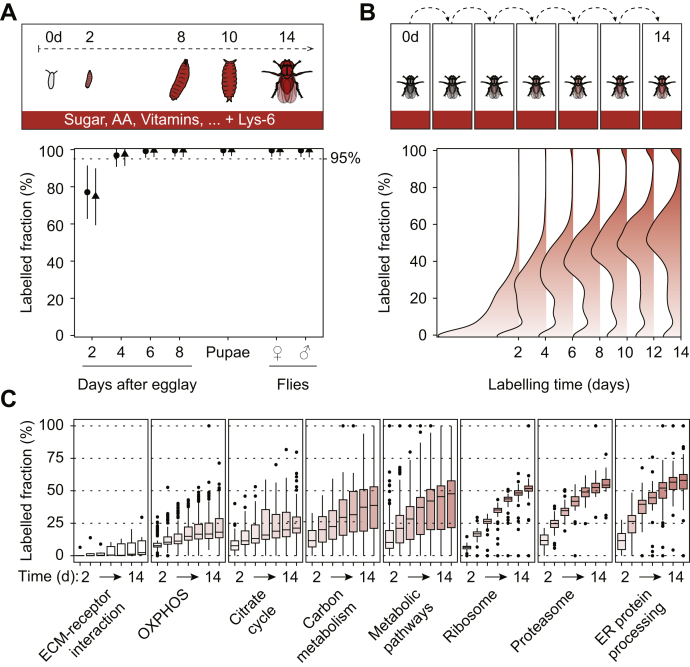
**Introducing the heavy label Lys-6 in *Drosophila* larvae and flies.***A*, protein incorporation rates of Lys-6 from unlabeled fertilized eggs to 1-day-old flies. *Dotted line* indicates 95% labeling fraction. Graph shows mean ± s.d. (n = 2). *B*, density curves of Lys-6 incorporation rates in adult fly proteins from 1-day-old unlabelld male flies, for 14 days, in 2-day intervals (pooled values, n = 2). *C*, labeling profiles as in (*B*) of significantly overrepresented KEGG functional categories (adj. *p* value < 0.05) by overrepresentation analysis (ORA) for proteins in each labeling quartile [0%–25%], [25%–50%], [50%–75%] and [75%–100%] of day 14, as indicated by dotted horizontal lines. Color coding according to median labeling fraction as in (*B*). Proteins in functional category are shown in *boxplots* with 25% and 75% percentile as the box with median, whiskers are ±1.5× interquartile range.

In order for SILAF to be used in a proteomic experiment, it is desirable to achieve full incorporation of the heavy amino acid in the proteome, and labeling rates above 95% are required to perform quantitative proteomic experiments (29). To measure incorporation efficiency of heavy lysine, we allowed unlabeled wild-type white Dahomey (*wDah*) flies, which had been grown on standard yeast food, to lay eggs for 8 h on holidic food containing heavy lysine. Labeling efficiency was assessed during *Dm* development in 2-day intervals by LC-MS/MS. Heavy lysine was incorporated rapidly, and already at 4 days after egg laying (dae), we reached 97.0% incorporation (Fig. 1*A*). By six dae, we reached 99.3% incorporation, achieving full labeling of larvae and flies.

As expected, incorporation rates of the heavy label were much lower in unlabeled adult flies transferred to heavy holidic food, reaching a maximum mean value of 45.0% labeling after 14 days (Fig. 1*B*), which likely originates from differences in the metabolic needs of larval and adult stages. We hypothesized that the slower turnover effectively represents the pulse phase of a pulse-chase experiment, indicating that SILAF can be used to determine protein turnover rates *in vivo*. Indeed, we observed that heavy-lysine incorporation during 2 weeks of labeling occurred in a nonuniform manner (Fig. 1*C*). Overrepresentation analysis against KEGG metabolic maps (30) showed that ribosome components acquired the label faster with median incorporation rates of 51.8%, while mitochondrial OXPHOS proteins ranged around 18.2% (supplemental Table S2).

### SILAF Is Compatible with Small-scale Peptide Fractionation

We tested the utility of holidic-food-based SILAF for quantitative proteomics. We first set out to evaluate its reproducibility and compared it with label-free quantification (LFQ). We obtained flies from larvae grown on either light or heavy holidic food, mixed the protein extracts, and performed single-shot LC-MS/MS analysis. We observed a high average (>0.95) Pearson’s correlation coefficient of the intensities of the heavy and light proteins (supplemental Fig. S1*E*), indicating high precision of the technique, low conversion of heavy lysine into other amino acids, and absence of artifacts related to labeling. We further achieved comparable correlation coefficient values and average standard deviation between replicates by LFQ on the light fraction of these samples (supplemental Fig. S1, *F* and *G*), indicating that the single-shot SILAF method is at least as powerful for quantification as label-free proteomics.

Quantification of proteins from flies is challenging due to their broad abundancy range independent of developmental stage (supplemental Fig. S2*A*). To assess the compatibility of SILAF with peptide fractionation, we grew flies from eggs on either heavy or light holidic food and performed small-scale high pH reversed-phase peptide fractionation on 20 μg of input peptides. The direct labeling-based comparison of male and female flies detected 4557 proteins and revealed profound differences in the abundance of a number of sex-related proteins (supplemental Fig. S2*B*; supplemental Table S3), including yolk sack proteins, which are exclusively expressed in the female fat body (31). A GSEA against gene ontologies revealed functional categories related to sex differentiation and mating (supplemental Fig. S2*C*). We further compared our results with those from Sury *et al.* (5) and observed good agreement in the level of regulation of sex-related proteins (supplemental Fig. S2*D*). Collectively, this data validates that SILAF is a highly efficient labeling method for *Drosophila* compatible with quantitative proteomic pipelines.

### The Holidic Diet Increases Protein Turnover

**Fig. 2:**
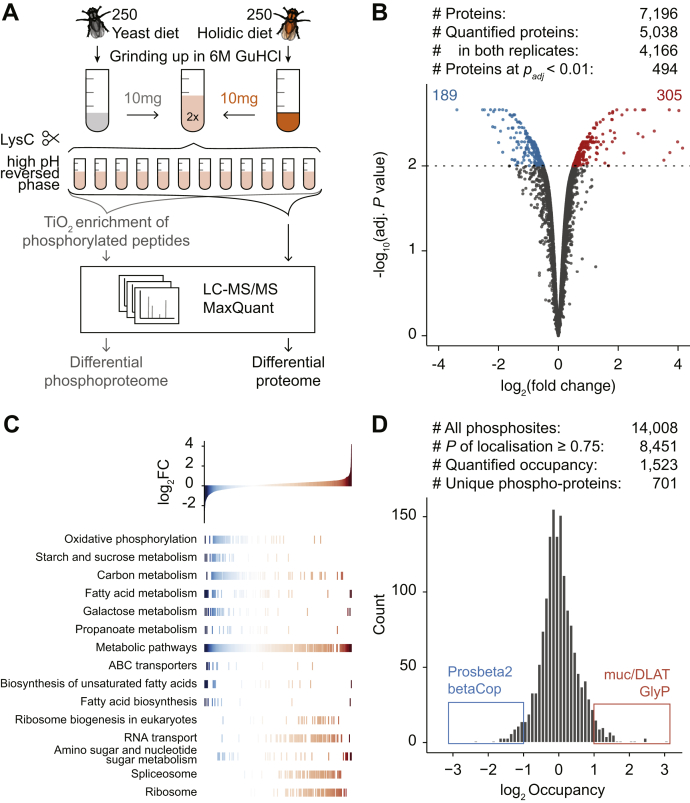
**Proteome and phosphoproteome quantification in holidic *versus* yeast food-grown flies.***A*, proteomic workflow to obtain a deep proteome and phosphoproteome dataset. Protein extracts, from flies grown on light yeast diet or heavy holidic diet, n = 2, were mixed, digested with endoproteinase Lys-C and separated by high pH reversed-phase chromatography. Twelve concatenated fractions were either analyzed directly by LC-MS/MS (proteome) or subjected to titanium dioxide enrichment (phosphoproteome). *B*, quantitative overview and volcano plot comparing holidic against yeast-food grown fly proteomes. *Dotted line* indicates the adj. *p* value threshold of 0.01, the number of significantly downregulated (*blue*) and upregulated (*red*) proteins is given in the *upper corners* (n = 2). *C*, gene set enrichment analysis of proteins shown in (*B*) against KEGG gene sets, ordered by rank and log_2_-fold change (FC) on x and y axis, respectively. Functional categories with FDR <0.05 are shown, each line represents one member of the category. *D*, quantitative overview and distribution of mean values of phosphosite occupancies (*lower panel*, n = 2).

Flies grown on this holidic food source are viable and fertile with life span comparable to maintenance on standard yeast-based compositions (14). It is well known that larvae grown on holidic food present with a prolonged developmental stage and pupate after approximately nine instead of 5 days after egg laying (dae) at 25 °C. To determine the proteome of SILAF—compared with yeast—food grown flies, we employed large-scale high pH reversed-phase fractionation (32) and TiO_2_-based phosphopeptide enrichment to perform quantitative proteome and phosphoproteome analysis on 1-day-old flies grown on either standard yeast food or on holidic food (Fig. 2*A*). We quantified 5038 proteins, and 494 proteins were significantly different at an adjusted *p* value <0.01 (Fig. 2*B*). Among these, the phosphoenolpyruvate carboxy kinase (Pepck), the rate-limiting enzyme in gluconeogenesis, was increased 3.7-fold on protein level in flies grown on holidic food (supplemental Table S4), while components of OXPHOS, carbohydrate, and fatty acid metabolism were decreased (Fig. 2*C*). Ribosomal components were upregulated (Fig. 2*C*), indicating an increased usage of amino acids over carbohydrates and fatty acids. We further detected 8451 phosphosites with a localization probability >0.75 and predominately present on serine residues, and SILAF allowed us to quantify the occupancy of 1523 phosphosites from 701 proteins (Fig. 2*D*; supplemental Fig. S3, *A*–*C*; supplemental Table S4). Proteins with upregulated occupancy included factors involved in glucose and glycogen metabolism such as the well-known activating pS15 on glycogen phosphorylase (UniProt ID Q9XTL) (33, 34), whereas components related to proteasome, OXPHOS, and ribosome showed reduced phosphorylation occupancy (Fig. 2*D*; supplemental Fig. S3, *D* and *E*). These findings together point toward a decreased usage of glucose in glycolysis, decreased fatty acid metabolism, and an increased turnover of amino acids due to the holidic food, feeding gluconeogenesis.

### Knockdown of DmLRPPRC1 in Flies Affects OXPHOS Protein Levels

**Fig. 3:**
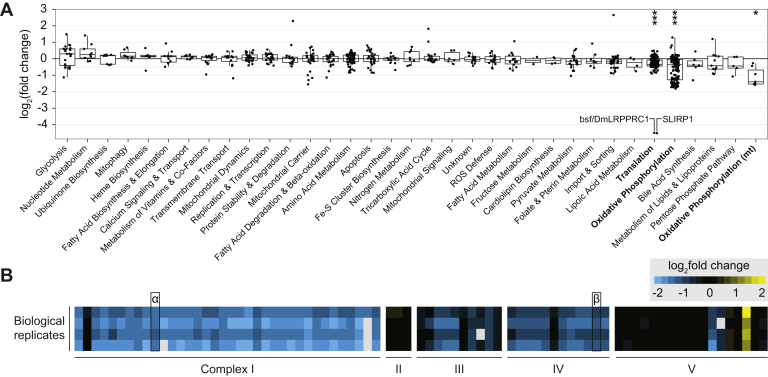
**Functional map of the mitochondrial proteome upon *DmLRPPRC1* knockdown.***A*, mitochondrial protein changes upon knockdown of LRPPRC in 9-day-old larvae compared with developmentally matched controls, divided into functional categories. *Boxplots* represent the categories’ 25% and 75% percentile as the box with median, whiskers are ±1.5× interquartile range. *Asterisks* and *bold labels* mark significantly changed categories at an adjusted *p* value below 0.05 (n = 4). *B*, heat mapped levels of detected category members “Oxidative Phosphorylation” and “Oxidative Phosphorylation (mt)” (*columns*) as in (*A*) of four biological replicates (*rows*) subsetted into their respective OXPHOS complexes I to V. *Gray boxes* indicate the absence of the measurement point. α- and β-marked *solid box*: NDUFB10 and NDUFA4.

In order to map and quantify phosphorylation sites of regulatory importance in the mitochondrial compartment, we applied SILAF to *DmLRPPRC1* knockdown (KD) larvae and enriched mitochondria by differential centrifugation (supplemental Fig. S4*A*). Loss of DmLRPPRC1 results in a severe combined OXPHOS deficiency due to mitochondrial mRNA instability, loss of polyadenylation, delayed larval development, and decreased life span (12). Mutations in the human homolog *LRPPRC* are implicated in a French–Canadian variant of Leigh syndrome (35). A total of 611 proteins were significantly changed in KD larvae (adj. *p* value < 0.01), corresponding to 12.5% of the total detected proteome, including nonmitochondrial background (supplemental Table S5). Using a curated set of *Dm* mitochondrial proteins (20), our data set covered 75.3% of the mitoproteome (supplemental Fig. S4*B*). “Translation” and “Oxidative Phosphorylation” were the only significantly altered functional categories in the mitochondrial compartment (Fig. 3*A*), supporting the notion that the chosen larval model and time point depict an early response with few secondary effects. This was confirmed by GSEA using proteins annotated as mitochondrial against KEGG gene sets, which highlighted ribosome and OXPHOS as the most significantly downregulated functional categories (supplemental Fig. S4*C*). We further subsetted to OXPHOS proteins and found a loss of subunits in complexes I, III, and IV, which all contain essential proteins that are encoded by the mitochondrial genome (mtDNA) (Fig. 3*B*). Complex II, which is entirely nuclear encoded, and V with two mitochondrially encoded subunits, were not affected. Likewise, we did not observe any significant changes across OXPHOS assembly factors and accessory factors (supplemental Fig. S4*D*). Notably, our *Dm* data corresponds well with observations made in the hearts of 7-week-old LRPPRC knockout mice (36) (supplemental Fig. S4*E*).

### The Mitochondrial Proteome is Hypophosphorylated

**Fig. 4:**
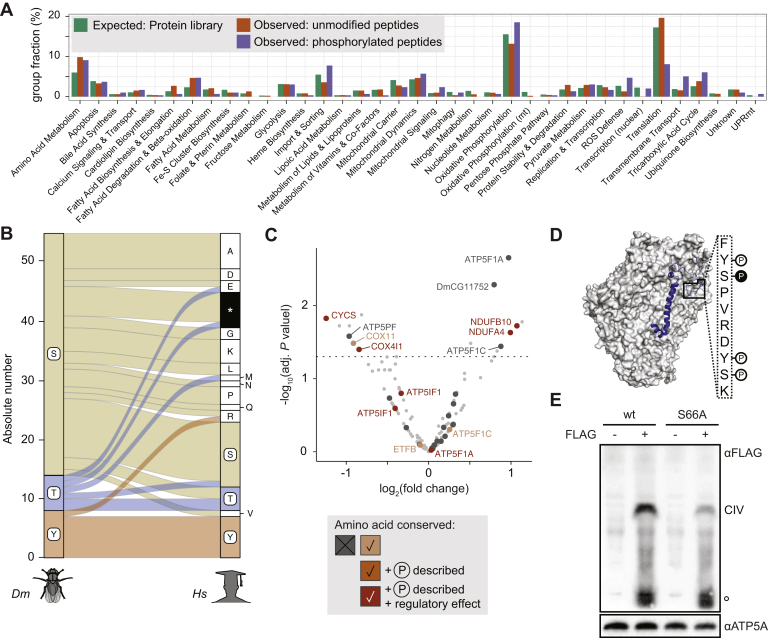
**Mitochondrial protein phosphorylation landscape upon *DmLRPPRC1* knockdown.***A*, relative number of phosphopeptide evidences in relation to the size of mitochondrial functional protein categories on protein and peptide level. *B*, evolutionary trace of phosphorylated OXPHOS serines, threonines, and tyrosines in *Dm* into corresponding *Hs* amino acids. ∗Residue absent. *C*, volcano plot of phospho-occupancy changes on mitochondrial proteins; *light gray*: all quantified phosphosites; *dark gray*, *orange*: OXPHOS components (see supplemental Fig. S5*B*; n = 4). *D*, position of NDUFA4 (*dark blue*) on complex IV (*gray*) with the phosphorylated peptide marked with a *rectangle*. The amino acid sequence within the marked stretch is depicted on the right (N-terminus at the top) and the position of the four possibly phosphorylated amino acids is indicated with *circles*. The *filled black circle* refers to pS66 and is the only significantly changed phosphosite in that peptide. *E*, western blot after blue native PAGE against NDUFA4 with or without a FLAG handle expressed in wild-type HEK293T cells. The band marked with “CIV” corresponds to fully assembled complex IV, ° is the free NDUFA4 subunit. ATP5A is used as a loading control. One representative image is shown (n = 3).

We next applied SILAF to identify the mito-phosphoproteome in *DmLRPPRC1* KD larvae. In agreement with human and mouse, we found the *Dm* mito-proteome hypophosphorylated on both the protein and peptide level compared with the nonmitochondrial background signal (supplemental Fig. S4*B*) (10, 11). Of 286 mitochondrial phosphosites, the functional category “translation” was especially low in phosphosites per peptide, whereas in relation to the total detectable mitochondrial peptidome, “Import & Sorting,” “ROS Defense,” and “Transmembrane Transport” were at a hyperphosphorylated state per peptide (Fig. 4*A*). Again, by combining quantitative changes in the phosphoproteome with changes in protein and peptide abundances, we were able to evaluate the occupancy changes of 697 (25.9%) out of 2686 detected phosphosites (supplemental Fig. S4*F*; supplemental Table S5). Of these, 59 were significantly changed (adj. *p* value < 0.05), with 19 predicted to be localized to mitochondria (supplemental Table S5). Interestingly, a conserved site on the DmLRPPRC1 interacting protein SRA stem-loop RNA-binding protein 1 (SLIRP1) was significantly decreased in occupancy (supplemental Fig. S5*B*). However, SLIRP1 protein levels decreased with DmLRPPRC1 (Fig. 3*A*), and it cannot be ruled out that the observed differences in phospho-occupancy are skewed due to the 20-fold reduction in protein levels.

We focused the analysis on the categories annotated as “oxidative phosphorylation” and “oxidative phosphorylation (mt),” whose peptide-to-phosphopeptide ratio ranged around the expected proportion within mitochondria. We detected a total of 55 phosphorylated residues on all OXPHOS complexes (supplemental Fig. S5*A*), predominantly on serine, but also on threonine and tyrosine. Serine residues had the highest mutation rate into other amino acids in corresponding conserved *H. sapiens* (*Hs*) proteins (Fig. 4*B*). Thereof, serine-to-alanine was the most likely exchanged and notably alanine is used as a similarly sized, but nonmodifiable substitute at serine positions in phosphorylation studies. In contrast, seven out of eight phospho-tyrosine residues were conserved from fly to *Hs*.

### NDUFB10 and NDUFA4 Contain Phosphorylation Sites That React to an OXPHOS Assembly Defect

Of the 55 detected OXPHOS phosphosites, we were able to quantify and statistically evaluate 33, of which 8 were significantly changed at an adj. *p* value <0.05 (Fig. 4*C*; supplemental Table S5). We reasoned that a phosphosite of profound structural and/or functional relevance would be conserved across species, while sites of minor functional importance, for instance, to fine-tune OXPHOS activity, might be lost or would have mutated together with the protein. We therefore narrowed down the data set by grading all sites according to the conservation status of the amino acid in *Hs* and/or previously observed phosphorylation in *Hs*. Interestingly, all sites on accessory subunits were conserved, five out of six had a previously identified phospho-group and two were shown to have a significant effect on OXPHOS activity (37, 38) (supplemental Fig. S5*A*). In contrast, only three of the 16 complex V phosphorylated residues are conserved to *Hs*.

Among the sites with significantly altered occupancy, we noted two phosphorylated and highly conserved residues, NDUFB10-pY126 and NDUFA4-pS66, both with a twofold occupancy increase despite 37% and 48% protein levels retained in *DmLRPPRC1* KD (Figs. 3*B* and 4*C*; supplemental Fig. S5*C*; supplemental Table S5). NDUFB10 (*Dm:* ND-PDSW) is situated in the PD membrane domain of complex I, facing the intermembrane space (39) (supplemental Fig. S5*D*). Y143, the conserved residue in HsNDUFB10, anchors NDUFB10 into ND4 in a cryo-EM structure of complex I (supplemental Fig. S5*E*) (25). ND4 is an mtDNA-encoded core component of complex I and thus a target of DmLRPPRC1 loss (12). The inaccessibility of the site to a kinase suggests that the phosphorylation is not dynamically regulated within the intact complex I structure, but rather happens during CHCHD4-dependent import (40). Potentially, pY143 maintains a free phosphorylated pool for regulatory purposes, targets the protein for degradation, or is required for insertion of the protein into the PD domain. We modeled the phospho-group onto Y143 using PyTMs (27) and did not find van der Waals straining altered between the modified and unmodified residue (164.89 *versus* 164.06, respectively). However, the potential phospho-group does not leave a cryoEM density trace and is only distantly coordinated by R245 on ND4 (supplemental Fig. S5*E*). Furthermore, the signal of the unmodified, but not the phosphopeptide decreases profoundly upon *DmLRPPRC1* KD (supplemental Fig. S5*F*), suggesting that only the unmodified protein is lost with the ND4 subunit (12) and a destabilized complex I.

The second hit, pS66 on NDUFA4 (*Dm*: ND-MLRQ), also termed COXFA4, is the 14th proposed subunit of complex IV (41). While its N terminus interacts with mtDNA-encoded COX1 and COX3, NDUFA4-S66 is situated on the C-terminal tail in the intermembrane space (Fig. 4*D*). Zong and colleagues demonstrated that NDUFA4 prevents dimerization of complex IV and fosters the formation of monomers and the supercomplex structure CI_1_CIII_2_CIV_1_ (26). It is tempting to speculate that S66 phosphorylation modulates complex IV dimerization and thus supercomplex formation. This could also explain the loss of high-molecular-weight bands in the BN-PAGE gels by Balsa and colleagues upon loss of NDUFA4 (42). In fact, HsNDUFA4-S66A-FLAG, which mimics the nonphosphorylated protein, associated less with mature complex IV upon overexpression in HEK293T cells compared with a cell line overexpressing wildtype NDUFA4-FLAG on blue native PAGE (Fig. 4*G*).

## Discussion

Label-free quantification by LC-MS/MS has made immense advances in recent years in accuracy and reproducibility, making many previously impossible analytical investigations now feasible (43). Additionally, it has led to a reduction in the dependency on techniques such as SILAC, as a quantitative view of the proteome can now also be obtained by LFQ. In fact, we find that SILAF is only marginally more reproducible than LFQ. However, for many applications that include fractionation or tracing, metabolic labeling with amino acid isotopes is the most accurate gold standard to date (44). SILAC in flies has been evaluated in several reports (5, 6, 45), which were all based on feeding flies with auxotrophic, Lys-8 labeled *Saccharomyces cerevisiae*. In principle, this allowed following a fly lab’s standard experimental routines, replacing normal with labeled yeast in a default medium. However, the amounts of yeast are difficult to obtain both technically and financially (6) and consequently experimental procedures were changed either toward feeding yeast paste or dried yeast (5, 45) or reduced yeast content in solid medium (6). Despite remaining costly (46), labeling ranged from 93.7% to 97.7%, and it had been shown that the reason for not achieving complete labeling has been yeast and not fly metabolism (5). Furthermore, as yeast had to be labeled first, comparable to *Escherichia coli* labeling in *Caenorhabditis elegans*-SILAC (47), amino acids are exposed to potential metabolic modifications in yeast cells. In fact, Chang *et al.* (6) observed undesirable conversion of both lysine and arginine into other amino acids in the fly SILAC proteome that negatively affects accurate quantification on peptide level.

Here, we present a method that takes advantage of a holidic food source for stable-isotope labeling of amino acids in fruit flies. SILAF circumvents undesirable amino acid conversion by directly feeding Lys-6 to flies instead of prelabeled yeast. Thereby, we achieve virtually complete labeling already in second instar larvae after 4 to 6 days. Notably, after initial setup of the method, the costs for 1 mg of fully lysine-labeled peptides, which equals material from one *Dm* vial, are below $15. This makes SILAF highly cost-effective compared with previous methods. A detailed protocol for preparing SILAF food is available in supplemental Table S1 and with notes and comments in ref. (48). The defined nature of the holidic food source, including eight essential amino acids, allows a great flexibility toward the metabolic label that shall be introduced. We have recently reported the application of a methyl-SILAC approach in *Dm* that uses mass shifts of methyl-groups originating from methionine-[methyl-^13^CH_3_] to increase the confidence in a protein residue modified by methylation (49, 50). This specified the mitochondrial protein methylproteome in *Dm* at 205 sites and suggests the contribution of methylation to regulation of mitochondrial function.

We demonstrate in three biological applications that SILAF can be used for high-accuracy quantitative proteomics. Firstly, in agreement with previous work (5), by applying small-scale fractionation of a female/male fly mix, we identified a number of differentially expressed proteins, including a prominent enrichment of yolk sack proteins 1, 2, and 3 in female flies, which have been shown to be exclusively present in the female fat body (31). Secondly, high pH reversed-phase fractionation and phosphopeptide enrichment gave deep insights into enzymatic processes that were changed upon feeding the holidic diet compared with yeast medium. Overlaying 5038 quantified proteins in one or both replicates with phospho-occupancies revealed an enzymatic shift toward increased amino acid usage, decreased carbohydrate and fatty acid metabolism with increased gluconeogenesis and glycogen breakdown. The two latter mechanisms point toward a low dietary availability of a carbon source (51). It appears that this metabolic shift is the cause for the later pupation of larvae reared on holidic food compared with yeast-based food. This has been observed in all holidic food sources that have been developed so far (52) and indicates the lack of one or several metabolites, which slows down larval development, rather than an unbalanced nutrient composition. Our data suggests that it is either a complex sugar branching off from glucose, as glycogen breakdown and gluconeogenesis are increased but not glycolysis. The holidic enzymatic set further supports notions of a fatty acid shortage. However, Piper *et al.* (13) reported that the missing compound(s) cannot be extracted with chloroform, most likely excluding lipid species. Yet, this does not limit the applicability of SILAF, providing experiments are well controlled. Indeed, we have used SILAF for immunoprecipitation approaches in the past with good results. Even more, the increased protein turnover at the ribosome and proteasome could be the reason for the high efficiency and complete labeling patterns observed.

As a third biological application, we quantified occupancies of mitochondrial protein phosphosites upon knockdown of *DmLRPPRC1*. This caused a rather specific decrease of proteins related to OXPHOS and translation that is in line with molecular data (12), contrasting complex patterns of mitochondrial protein phosphorylation in obese mice (7, 10). We observed that phosphorylation sites on the fly mitochondrial proteome are underrepresented, which is in line with data from a human cell line (11) and mice (10). Several kinases and phosphatases have been localized to mitochondria as reviewed in (9), indicating that phosphorylation within mitochondria might be an important mechanism for regulating protein activity, half-life, or complex formation (53). Additionally, components of OXPHOS have been found phosphorylated for regulatory purposes; such as the PKA-dependent phosphorylation of ATPase Inhibitory Factor 1 (38), cytochrome c (37), and subunits of complex IV (54). This was well reproduced in our data, and phosphorylation occupancies decreased significantly on both cytochrome C and COX4I1 upon *DmLRPPRC1* knockdown.

We detected regulated sites on NDUFB10 and NDUFA4/COXFA4, which are both conserved from fly to human on amino acid, as well as posttranslational modification level (24, 55, 56). NDUFB10 is a vital subunit of complex I and a patient with compound heterozygous mutations in NDUFB10 presented with symptoms of mitochondrial disease, defects in late stage complex I assembly, and reduced complex I activity (40). The molecular mechanisms regulating NDUFB10 phosphorylation are unclear. NDUFB10 was found to be a target of the proto-oncogene tyrosine kinase Src, and inhibition of Src correlated with decreased OXPHOS activity (57). Y143 might be the Src target location; however, a set of molecular studies are required to confirm this and complement this proteomic screen.

## Data Availability

The mass spectrometry proteomics data and search engine results have been deposited to the ProteomeXchange Consortium *via* the PRIDE partner repository (58) with the data set identifier PXD009990. Spectra are deposited at MS-Viewer (59) with the search keys ql09hmiwx3 (methodological setup, underlying Fig. 1 and supplemental Fig. S1), xt460opfcy (female/male comparison, supplemental Fig. S2), hpvyjbouev (holidic *versus* yeast food proteome and phosphoproteome, Fig. 2 and supplemental Fig. S3), and bifkhsj6f9 (knockdown of *bsf/DmLRPPRC1*, Figs. 3 and 4, supplemental Figs. S4 and S5).

## Supplemental Data

This article contains supplemental data (14, 21).

## Conflict of interest

The authors declare no conflict of interest.
