# Supplementary material for: Stable Isotope Labeling of Amino Acids in Flies (SILAF) Reveals Differential Phosphorylation of Mitochondrial Proteins Upon Loss of OXPHOS Subunits
Source: Mol Cell Proteomics. 2021 Feb 25;20:100065. doi: 10.1016/j.mcpro.2021.100065 (PMC8050774; doi:10.1016/j.mcpro.2021.100065)
Supplement: Supplemental Figures S1–S5 [file mmc1.pdf]

Supplemental Figures S1 - S5

for

Direct proteome labelling in fruit flies with SILAF reveals differential phosphorylation of mitochondrial proteins upon loss of OXPHOS subunits

Florian A. Schober, Ilian Atanassov, David Moore, Javier Calvo-Garrido, Marco F. Moedas, Anna Wedell, Christoph Freyer, Anna Wredenberg

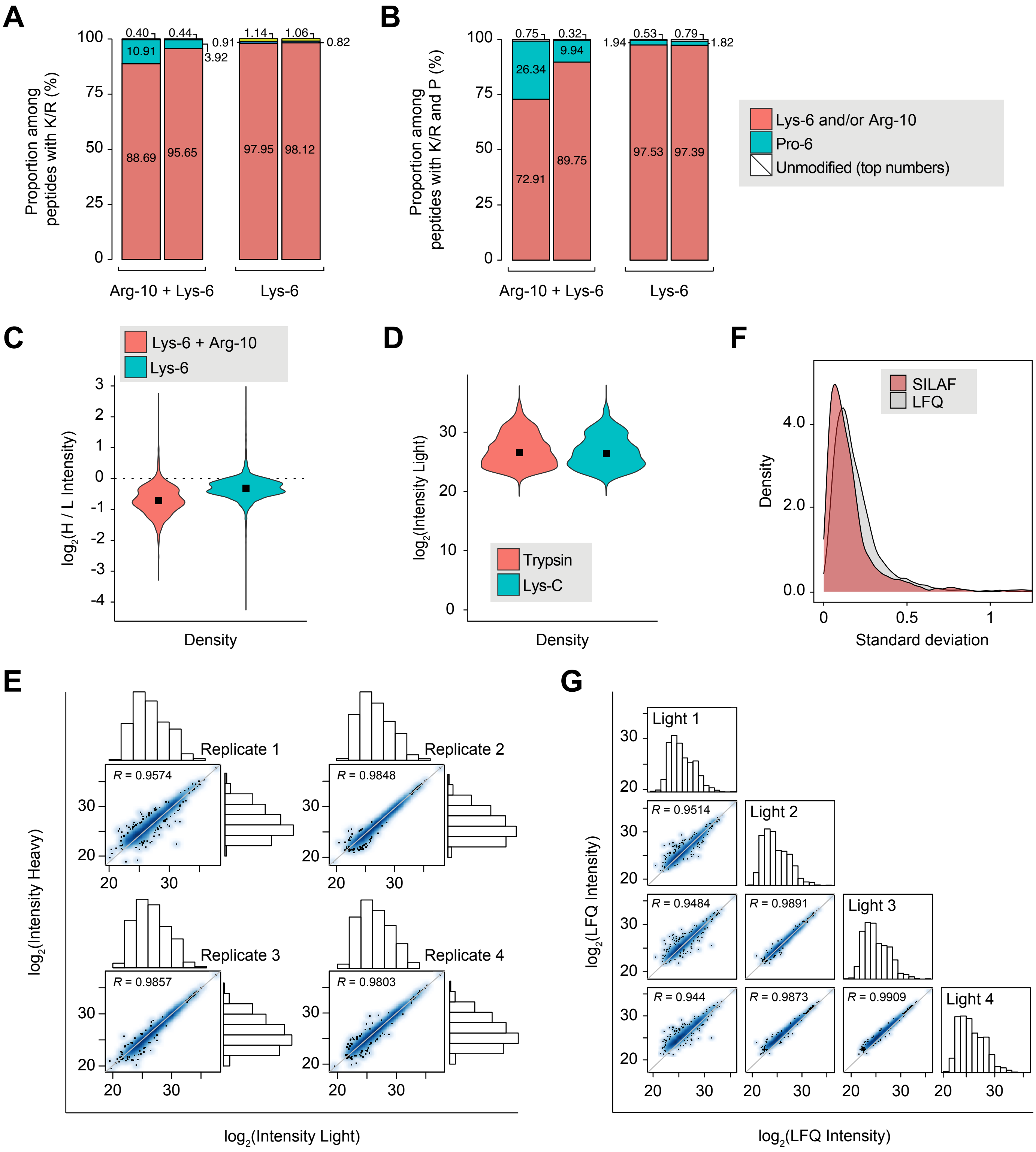

A
B
C
D
E
F
G
Proportion among peptides with K/R (%)
Proportion among peptides with K/R and P (%)
Arg-10 + Lys-6
Lys-6
Lys-6 and/or Arg-10
Pro-6
Unmodified (top numbers)
88.69
10.91
0.40
95.65
0.44
0.91
3.92
97.95
1.14
98.12
1.06
0.82
72.91
26.34
0.75
89.75
9.94
0.32
1.94
97.53
0.53
97.39
0.79
1.82
Lys-6 + Arg-10
Lys-6
log2(H / L Intensity)
Density
Trypsin
Lys-C
log2(Intensity Light)
SILAF
LFQ
Standard deviation
Replicate 1
Replicate 2
Replicate 3
Replicate 4
R = 0.9574
R = 0.9848
R = 0.9857
R = 0.9803
log2(Intensity Heavy)
log2(Intensity Light)
Light 1
Light 2
Light 3
Light 4
R = 0.9514
R = 0.9484
R = 0.9891
R = 0.944
R = 0.9873
R = 0.9909
log2(LFQ Intensity)

Figure S1

FIG. S1. **Quality control of the SILAF method.** *A*, Proportion of labelled peptides in flies (n = 2) grown on medium with either Arg-10 and Lys-6 (left two bars) or Lys-6 (right two bars), proportion of unlabelled and Pro-6 containing peptides among peptides containing lysine or arginine. Two replicates are shown each. *B*, Data as in (A) for all peptides that contain proline, and lysine or arginine. *C*, The double Lys-6/Arg-10 label causes a partial loss in apparent intensity of identified peptides in comparison to Lys-6. Light (L) peptides were derived from *wDah* grown on holidic diet (n = 3). The heavy (H) fraction was labelled with Lys-6 and Arg-10 and digested with trypsin, or Lys-6 only and digested with Lys-C. The graph is based on non-normalized intensity values. Samples as of (A). Black square: median. *D*, Lys-C peptides have a similar abundance profile than tryptic peptides. The plot shows non-normalized intensity of light SILAF fractions derived from light/heavy protein mixes from holidic food, digested either with trypsin or Lys-C (n = 3). Black square: median. *E*, Correlation scatter plots of non-normalized intensity values of a holidic SILAF diet proteome heavy against light with respective Pearson's correlation coefficients and 45-degree linear regression lines (n = 4). *F*, Standard deviation per quantified protein of normalized H/L ratios of a holidic diet proteome heavy against light SILAF food and label-free quantification intensities of the light fraction of the same sample shown in (E) (n = 4 per density plot). Only proteins that were detected in three out of four samples were included. *G*, LFQ quantifications with respective Pearson's correlation coefficients *R* and 45-degree linear regression lines. Samples as in (F) (n = 4).

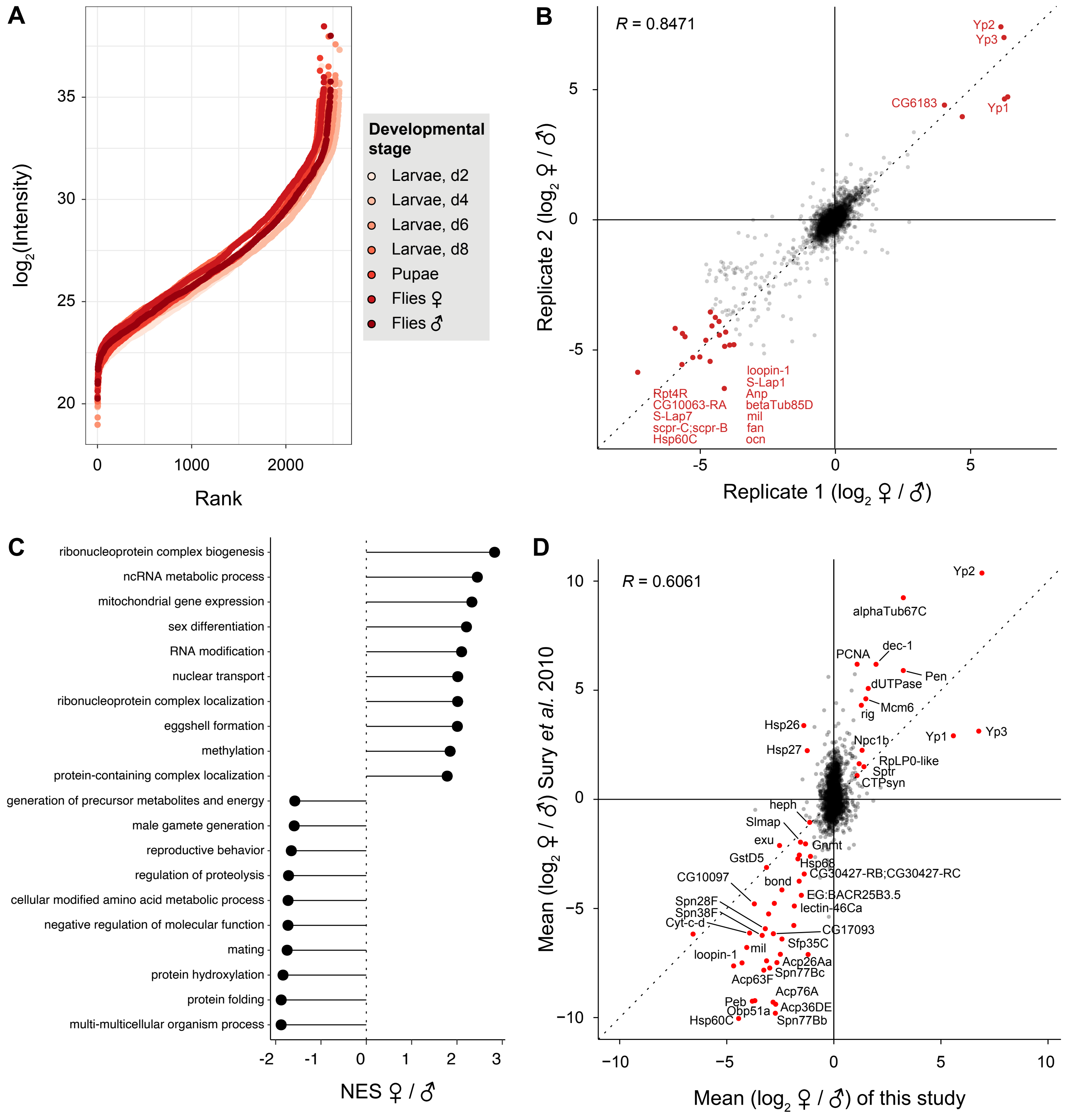
A
B
C
D
log2(Intensity)
Rank
Developmental stage
Larvae, d2
Larvae, d4
Larvae, d6
Larvae, d8
Pupae
Flies ♀
Flies ♂
R = 0.8471
Replicate 2 (log2 ♀ / ♂)
Replicate 1 (log2 ♀ / ♂)
Yp2
Yp3
Yp1
CG6183
Rpt4R
CG10063-RA
S-Lap7
scpr-C;scpr-B
Hsp60C
loopin-1
S-Lap1
Anp
betaTub85D
mil
fan
ocn
ribonucleoprotein complex biogenesis
ncRNA metabolic process
mitochondrial gene expression
sex differentiation
RNA modification
nuclear transport
ribonucleoprotein complex localization
eggshell formation
methylation
protein-containing complex localization
generation of precursor metabolites and energy
male gamete generation
reproductive behavior
regulation of proteolysis
cellular modified amino acid metabolic process
negative regulation of molecular function
mating
protein hydroxylation
protein folding
multi-multicellular organism process
NES ♀ / ♂
R = 0.6061
Mean (log2 ♀ / ♂) Sury et al. 2010
Mean (log2 ♀ / ♂) of this study
Yp2
alphaTub67C
PCNA
dec-1
Pen
dUTPase
Mcm6
rig
Hsp26
Yp1
Yp3
Npc1b
Hsp27
RpLP0-like
Sptr
CTPsyn
heph
Slmap
exu
Gnmt
GstD5
Hsp68
CG30427-RB;CG30427-RC
CG10097
bond
EG:BACR25B3.5
Spn28F
Spn38F
lectin-46Ca
Cyt-c-d
CG17093
Sfp35C
mil
loopin-1
Acp26Aa
Spn77Bc
Acp63F
Acp76A
Peb
Obp51a
Acp36DE
Hsp60C
Spn77Bb

Figure S2

Fig. S2. **SILAF based comparison of female and male fly proteomes.** *A*, Dynamic range of non-normalized protein intensities in relation to developmental stages. Ranks are assigned by increasing intensity order (n = 1). Data as in Figure 1A, Lys-6 labelling only. d, developmental day. *B*, Overlap of log_2-transformed ratios of female to male proteomes as two biological SILAF replicates. Replicate 2 was performed with inverted labels. Red dots show targets with an absolute log_2-fold change of more than 4 in both replicates. *C*, Gene set enrichment analysis (n = 2) against Gene Ontology terms within "Biological Processes" of all proteins in (B). Top 20 terms ranked by FDR plotted with their normalized enrichment score (NES). *D*, Comparison of the results of this study obtained with SILAF to previous data that was generated from flies grown on labelled yeast (5). *R* is Pearson's correlation coefficient.

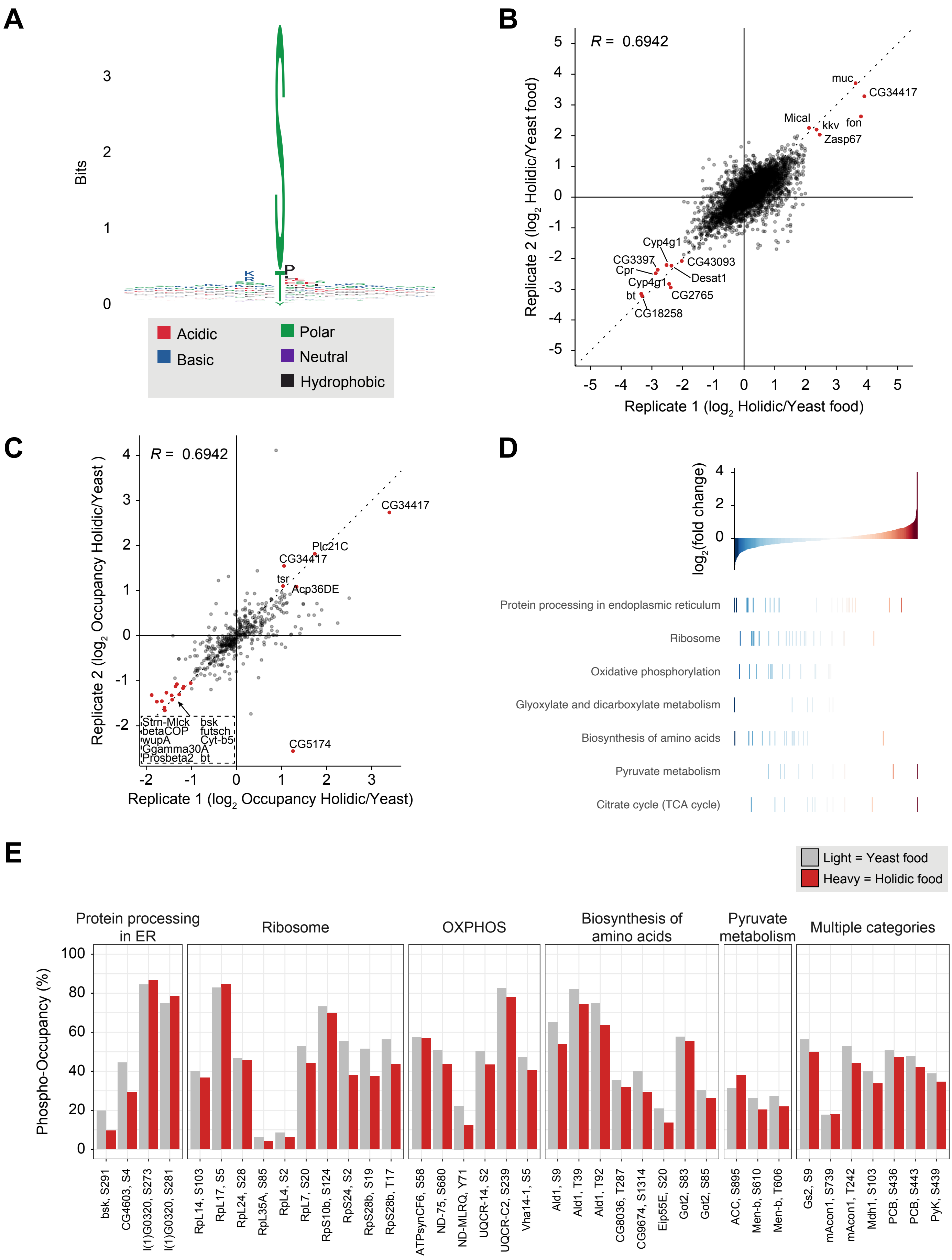

A
Bits
3
2
1
0
K
R
P
Acidic
Basic
Polar
Neutral
Hydrophobic
B
R = 0.6942
Replicate 2 (log2 Holidic/Yeast food)
Replicate 1 (log2 Holidic/Yeast food)
5
4
3
2
1
0
-1
-2
-3
-4
-5
muc
CG34417
Mical
kkv
fon
Zasp67
Cyp4g1
CG3397
Cpr
CG43093
Desat1
Cyp4g1
bt
CG2765
CG18258
C
R = 0.6942
Replicate 2 (log2 Occupancy Holidic/Yeast)
Replicate 1 (log2 Occupancy Holidic/Yeast)
4
3
2
1
0
-1
-2
CG34417
Plc21C
CG34417
tsr
Acp36DE
CG5174
Strn-Mlck
bsk
betaCOP
futsch
wupA
Cyt-b5
Ggamma30A
Prosbeta2
bt
D
log2(fold change)
4
2
0
Protein processing in endoplasmic reticulum
Ribosome
Oxidative phosphorylation
Glyoxylate and dicarboxylate metabolism
Biosynthesis of amino acids
Pyruvate metabolism
Citrate cycle (TCA cycle)
E
Light = Yeast food
Heavy = Holidic food
Phospho-Occupancy (%)
100
80
60
40
20
0
Protein processing in ER
Ribosome
OXPHOS
Biosynthesis of amino acids
Pyruvate metabolism
Multiple categories
bsk, S291
CG4603, S4
l(1)G0320, S273
l(1)G0320, S281
RpL14, S103
RpL17, S5
RpL24, S28
RpL35A, S85
RpL4, S2
RpL7, S20
RpS10b, S124
RpS24, S2
RpS28b, S19
RpS28b, T17
ATPsynCF6, S58
ND-75, S680
ND-MLRQ, Y71
UQCR-14, S2
UQCR-C2, S239
Vha14-1, S5
Ald1, S9
Ald1, T39
Ald1, T92
CG8036, T287
CG9674, S1314
Eip55E, S20
Got2, S83
Got2, S85
ACC, S895
Men-b, S610
Men-b, T606
Gs2, S9
mAcon1, S739
mAcon1, T242
Mdh1, S103
PCB, S436
PCB, S443
PyK, S439

Figure S3

Fig. S3. **Differential phosphoproteome of holidic versus yeast food-grown flies.** *A*, Phospho-site enrichment (8373 sequences). *B*, Overlap of heavy (H, holidic diet) over light (L, yeast diet) ratios and, *C*, phospho-occupancies of phospho-peptides in two replicates. *R* is Pearson's correlation coefficient. Proteins with an absolute $\log_2$-fold change > 2 (B) or > 1 (C) are plotted in red and labelled with gene symbols. *D*, Gene set enrichment analysis of phospho-sites occupancies shown in (C) against KEGG gene sets (n = 2). Significant categories ($P < 0.05$) are shown. Each line represents one member of a functional category. Proteins are ordered by rank and $\log_2$-fold change on x and y axis, respectively. *E*, Occupancies of targets in the categories listed in (D). Only proteins with a quantified occupancy > 0 in both replicates are shown as mean value for each labelling state.

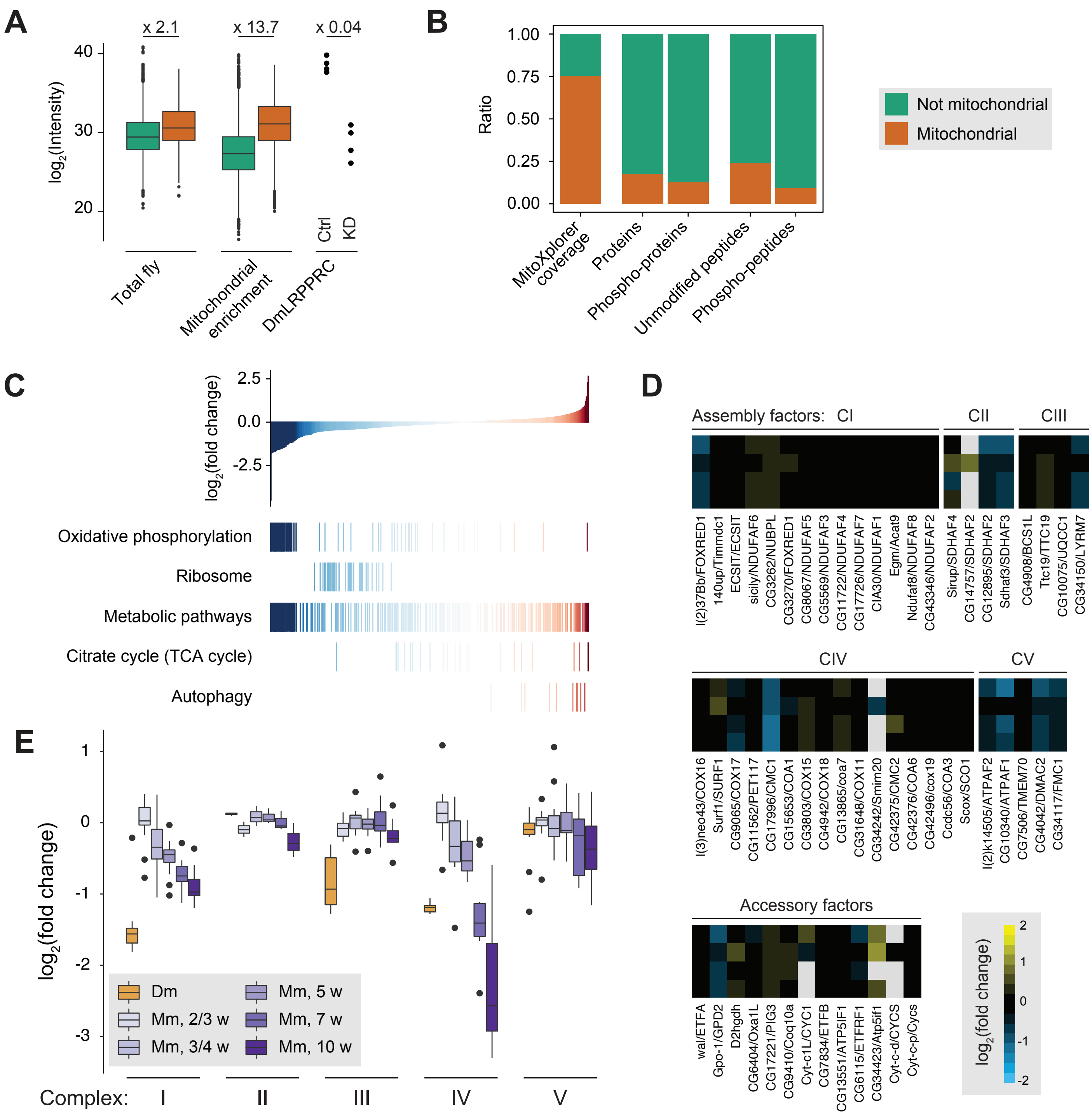
A
x 2.1
x 13.7
x 0.04
log2(Intensity)
Total fly
Mitochondrial enrichment
DmLRPPRC
Ctrl
KD
B
Ratio
MitoXplorer coverage
Proteins
Phospho-proteins
Unmodified peptides
Phospho-peptides
Not mitochondrial
Mitochondrial
C
log2(fold change)
Oxidative phosphorylation
Ribosome
Metabolic pathways
Citrate cycle (TCA cycle)
Autophagy
D
Assembly factors: CI
CII
CIII
CIV
CV
Accessory factors
log2(fold change)
E
log2(fold change)
Dm
Mm, 2/3 w
Mm, 3/4 w
Mm, 5 w
Mm, 7 w
Mm, 10 w
Complex: I II III IV V

Figure S4

Fig. S4. **Mitochondrial proteome upon DmLRPPRC1 knockdown.** *A*, Left two panels: Distribution of non-normalized intensity values in relation to proteins annotated as mitochondrial in fractionated total fly protein extracts used in Fig. 2, or enriched mitochondrial proteins used in Fig. 3. Right panel: Non-normalized intensity values of DmLRPPRC1 in controls or KD. Fold changes on top indicate the ratio of compared medians. Boxplots indicate 25% and 75% percentile and median as the box, whiskers are ±1.5x interquartile range. *B*, Left: Coverage ratio of proteins identified in this study against proteins with mitochondrial annotation. Four panels on the right: Fraction of proteins, peptides, phosphoproteins and phospho-peptides annotated as mitochondrial against the full dataset. *C*, Gene set enrichment analysis of mean fold changes upon *DmLRPPRC1* KD against KEGG gene sets (FDR < 0.05). Each line represents one member of a functional category. Proteins are ordered by rank and log_2-fold change on x and y axis, respectively. *D*, Heat mapped levels of OXPHOS assembly and accessory factors of four biological replicates (rows) subsetted into function and complex (columns) with fly gene names/ human ortholog symbols. *E*, Grouped log_2-tranformed fold changes of OXPHOS complex subunits in this *Drosophila* study (*Dm*, yellow) and a comparable mouse study (36) (*Mm*, shades of blue). The age of mice is given in the legend. w, weeks.

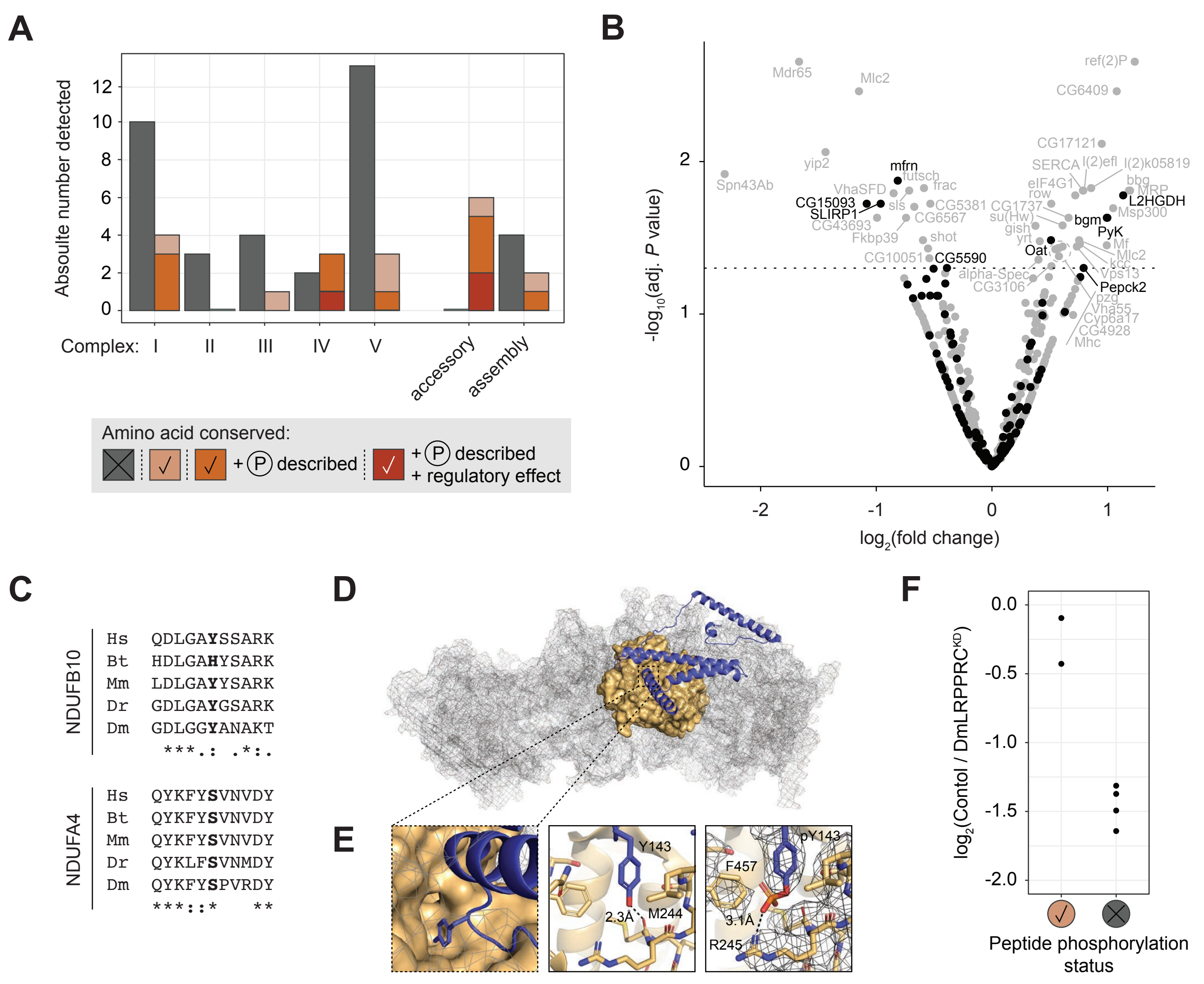

FIG. S5. **Mitochondrial phosphoproteome upon DmLRPPRC1 knockdown.** *A*, Conservation status of 55 detected OXPHOS phospho-sites by function and complex. *B*, Volcano plot of phospho-occupancy changes of KD against controls, excluding OXPHOS members (see Fig. 4C). Black: annotated mitochondrial proteins in MitoXplorer (35); Grey: not annotated as mitochondrial. *C*, Homologous peptide stretches covering phosphorylated Y126 on NDUFB10 and S66 on NDUFA4 (bold). * full conservation; : strong similarity; . weak similarity. *D*, Position of NDUFB10 (blue) in human complex I as seen from the intermembrane space; yellow: ND4. *E*, Anchoring of NDUFB10 into ND4 by tyrosine 143 as seen from the intermembrane space (left), in absence (middle) and presence (right, including cryo-EM density mesh) of a modelled phospho group. Black dotted lines indicate distances. *F*, Fold change of the Y126-phosphorylated and unphosphorylated DmNDUFB10 peptide in KDs against controls. Each dot represents one replicate, values are not normalized.
